# Optimizing practice scheduling requires quantitative tracking of individual item performance

**DOI:** 10.1038/s41539-020-00074-4

**Published:** 2020-10-15

**Authors:** Luke G. Eglington, Philip I. Pavlik Jr

**Affiliations:** Institute for Intelligent Systems, University of Memphis, Memphis, TN USA

**Keywords:** Education, Education, Human behaviour

## Abstract

Decades of research has shown that spacing practice trials over time can improve later memory, but there are few concrete recommendations concerning how to optimally space practice. We show that existing recommendations are inherently suboptimal due to their insensitivity to time costs and individual- and item-level differences. We introduce an alternative approach that optimally schedules practice with a computational model of spacing in tandem with microeconomic principles. We simulated conventional spacing schedules and our adaptive model-based approach. Simulations indicated that practicing according to microeconomic principles of *efficiency* resulted in substantially better memory retention than alternatives. The simulation results provided quantitative estimates of optimal difficulty that differed markedly from prior recommendations but still supported a desirable difficulty framework. Experimental results supported simulation predictions, with up to 40% more items recalled in conditions where practice was scheduled optimally according to the model of practice. Our approach can be readily implemented in online educational systems that adaptively schedule practice and has significant implications for millions of students currently learning with educational technology.

## Introduction

**Fig. 1:**
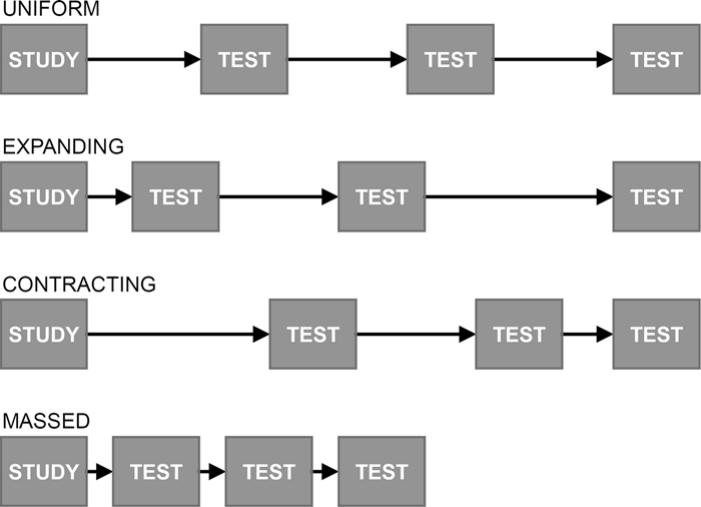
Common conventional spacing schedules. Several conventional spacing schedules.

There are large potential benefits to applying findings from the cognitive science of learning to educational contexts. Two especially promising findings are spacing and retrieval practice (testing). Spacing practice trials across time has been shown to benefit memory^1,2^. Testing has also been shown to benefit memory, relative to restudying^3,4^. Taken together, spaced retrieval practice has been shown to provide substantial benefits to later memory^5^. Successfully integrating these findings into educational technology could benefit millions of students who currently learn in online educational systems. However, implementation of these findings has been elusive—research has been unclear regarding exactly how much and when to practice specific information. We demonstrate how standard methods to uncover optimal practice schedules are confounded by fundamental characteristics of memory and that any single conventional schedule (see Fig. 1) applied to a set of items will be inherently suboptimal. We provide evidence that adaptive schedules lead to superior performance, better accommodate item and learner variability, and are implementable with a mixture of computational memory models and relatively simple economic decision rules.

Researchers have explored the effects of various spacing intervals on memory^6–8^, frequently while enforcing fixed trial durations and numbers of trials. Figure 1 depicts several popular spacing schedules compared in the literature: the inter-trial interval could be a fixed value (first row of Fig. 1), gradually increase over time (second row), or decrease over time (third row). Those three cases in Fig. 1 have the same average interval, but the distribution of the spacing could influence performance and difficulty. For instance, the longer initial retention interval for the uniform condition (Fig. 1) may increase potential learning but also entails a higher risk of retrieval failure. More generally, there is a trade-off between the potential gain from more spacing and the risk of retrieval failure. This trade-off is relevant to a popular theoretical explanation of the spacing effect named *study-phase retrieval*, which states that retrieving memories of previous exposures to an item strengthens the memory trace^9,10^. Retrieval difficulty is thought to influence the benefit—more difficult retrieval is thought to better strengthen a memory but is riskier since it may fail.

There is also empirical evidence that practice can *slow* forgetting^11^, which implies that as practice accumulates retrieval becomes easier. This finding of slowed forgetting, in combination with study-phase retrieval theories, implies that gradually increasing difficulty (via spacing) may balance difficulty and lead to better memory via an expanding schedule. Surprisingly, research findings have been more mixed than may have been predicted by the theories and empirical results described above. For instance, Landauer and Bjork^12^ found that an expanding schedule of test trials without feedback resulted in better memory at a final test than both uniform and contracting schedules. In contrast, Karpicke and Roediger^13^ showed that providing feedback could lead to a benefit of uniform scheduling. Recently, Kang et al.^14^ found that expanding intervals provided higher average recall probability across multiple sessions but equal memory to uniform intervals at a final test.

Some heuristic schedules that adapt to item-level differences have been evaluated, such as dropping an item from practice once it has been answered correctly a specific number of times^15^. These methods offer the possibility to shift practice time from items that are easier (have been recalled successfully) to those that are more difficult. Using vocabulary word pairs for learning materials, Pyc and Rawson compared the memorial benefits of conventional spacing schedules (e.g., a uniform schedule) to those that dropped items from practice after being successfully recalled. They found that a drop schedule in which word pairs were spaced by 23 intervening pairs and were dropped after one successful retrieval resulted in superior final test memory than conventional schedules. However, variation in item difficulty and participant performance make it unlikely that the number of intervening items or number of correct answers to allow before dropping (1) would be generally optimal. Drop heuristics are also silent regarding when an item should be reintroduced after being dropped from practice.

A promising approach that values efficiency is the Region of Proximal Learning (RPL) framework. RPL recommends that students should choose to practice items that are “..slightly beyond the individual’s grasp”^16^. Relatedly, Tullis et al.^17^ showed that allowing students to choose what to study provided significant learning benefits. A challenge for many of these frameworks is that it is not clear when previously practiced items should be reintroduced. If left up to the student, then their subjective judgments would need to be well calibrated. Allowing student choice may also introduce additional time costs: the candidate study items would need to be presented in some fashion for one to be selected. A related challenge with RPL, common to many approaches, is one of implementation. Time cost is considered important and used to justify the practice of easier items^16^, but difficulty and time costs are not quantified, which makes it unclear how to apply the principles broadly. Additionally, whether easier items are more efficient depends on the learning gains from successes and the informativeness of feedback when failures occur. Considering time cost could reveal in some cases that easy items are preferable (when success causes strong learning and feedback is more time-consuming) but in others that more difficult items should be practiced instead (if feedback after failures is informative and relatively quicker).

Many proposed practice schedules manipulate difficulty in search of improved learning outcomes. However, an under-appreciated consequence of varying difficulty is the effect of difficulty on the time needed to complete the task, since students and educators are limited in the time they can spend being instructed or instructing. Both learning gains and time costs are objectively important measures. Thus gains in learning should be valued in relation to their time cost, and costly learning methods are relatively less valuable than faster methods. Considering both cost and gain leads to practice decisions that are sensitive to the value of the item (in terms of learning gains per second). If practice is too difficult, learners may take longer to recall when correct, and they will more consistently fail, which may be time-consuming due to review costs and lead to less learning per second.

Microeconomics provides the conceptual tools to consider the gain from a trial along with its cost (in time); maximizing gain and minimizing costs is a common concern in economics research. In short, determining the optimal practice schedule is a fundamentally economic problem: find the maximally efficient level of difficulty that provides the most gains in learning per unit of time. To motivate simulations and experiments, below we describe the important relationship between practice and latency. We then describe the economic principles that motivate scheduling according to efficiency, in contrast with conventional practice schedules that frequently have focused on gain unconditional on time cost.

There is strong evidence that as practice accumulates, the time it takes for a participant to respond decreases (response time, henceforth RT). Correct trials are also frequently faster than incorrect trials, especially if feedback is required after errors^18^. As Kahana and Loftus^19^ describe, correctness and RT are typically considered in isolation even when they can be mutually informative. As we will show below, using only one measure to determine practice scheduling can be misleading. The reliable relationship between practice and RT has been shown in many contexts and paradigms^20,21^. Many researchers have shown strong relationships between practice and RT with declarative concepts. Anderson et al.^22^ demonstrated this consistent relationship with participants learning declarative concepts and rule application. Anderson^23^ showed that RT can be accurately modeled as an exponentially decaying function of memory activation. Similarly, Newell and Rosenbloom^24^ showed that RT can follow a power function of prior practice attempts.

The importance of the practice–RT relationship remains regardless of whether the relationship follows a power law or an exponential decay curve, the critical point is that the *efficiency* of practice (in terms of time) is related to when and how much practice has already occurred. The importance of the efficiency of practice has been mentioned in research concerned with improving learning outcomes with study techniques such as testing and spacing, although RT has not usually influenced interpretation when comparing conditions^15,25–27^. To optimize a schedule of practice gain relative to time spent, the relationship between practice latency and gain must be included in the analysis. Longer spacing intervals may increase the effect size of spacing, but if a wide interval takes substantially longer overall than a narrower interval, it may be a suboptimal use of a learner’s time. For instance, Yan et al.^28^ found that a very wide spacing interval provided comparable (or slightly higher) memorial benefits than a narrower spacing schedule. However, RTs during practice were significantly longer in the wider spacing schedule, resulting in the wider spacing interval being slightly *less* efficient per second of practice.

Although the primary measure of interest in many studies on spacing is final test performance, correctness *during* practice is also critical because it is an important determinant of efficiency. For instance, while feedback after failed retrieval attempts increase the benefit of testing^29^, this feedback takes time. However, If a participant is correct, feedback may be unnecessary^30^. Thus, in the context of vocabulary learning and other paradigms where processing and responding to the stimulus are fast, correct trials are often a faster and relatively more efficient use of a learners’ time.

In sum, if the efficiency of a practice attempt is defined as *the gain in retrievability at some final test divided by the time cost of the current practice*, there is strong theoretical reasoning and empirical evidence that the efficiency of an individual practice attempt will vary as practice accumulates for most learning tasks, depending on the number of prior practice attempts and their temporal spacing.

When using a model of practice to schedule learning activities, the goal is to use model predictions to make pedagogical decisions that maximize learning. One approach would be to choose the item which will provide the most gain (e.g., in the probability of recall) at a final test. However, there are several ways to measure gain with different shortcomings. For instance, percent gain^31^ or logit gain could be maximized. An efficiency score^32^ could also be composed from either as a measure of gain. In economics, this choice of the gain metric is a choice about how to measure utility (or value) in the domain. Utility in learning represents some way to order our preferences for learning events (practice trials) such that we can compare which learning events are more useful than other learning events. Adapting this term to education, we need a measure of learning value to compare different possible outcomes of instruction to guide practice scheduling decisions.

However, choosing a gain metric without considering time costs can result in inefficient practice. For instance, if maximal percentage gain in recall probability determines item selection, more difficult (lower probability items) will be chosen, causing higher error rates and increased time per trial. Selection focused on higher error rates tends to be inefficient unless time costs are ignored^18,33^.

Considering both variable cost and valuation leads to an alternative formulation of gain—the *efficiency* of practice. Computing the efficiency of practice is an inherently economic calculation in which the expectancy of the choices’ outcomes are relevant. Learning tasks are frequently tests where the attempt can fail or succeed, and each of these outcomes has different consequences. Calculating efficiency requires a measure of learning utility and of the cost, typically in terms of time. An economic interpretation of practice utility could be the following:

1 $$U = (p)\frac{{\rm correct}\;{\rm gain}}{{\rm correct}\;{\rm cost}} + (1 - p)\frac{{\rm incorrect}\;{\rm gain}}{{\rm incorrect}\;{\rm cost}},$$

where *p* is the probability of correctness. Gains and costs for correct and incorrect trials can be quite different, and thus practice difficulty is highly relevant to optimizing practice scheduling.

If the success or failure of a trial influences efficiency, at what probability of recall (difficulty) should items be practiced to maximize efficiency? In the present study, we aimed to find this optimal efficiency threshold (OET) via simulation, which requires a model that can accurately estimate the probabilities of recall and gain from practice and spacing. We subsequently tested the results of the simulation with an experiment. Next we describe candidate models as well as how they can be used to accommodate our efficiency-oriented approach.

Several excellent computational models of memory have been proposed^34–36^. These models have attempted to account for effects of repeated practice, spacing, forgetting (interference), and the cross-over interaction between practice spacing and the retention interval between the practice session and the final test^36,37^. The models described below track the most critical patterns found in studies manipulating practice and spacing.

Pavlik and Anderson^18,38^ attempted to account for the complex interactions associated with spacing practice over time by assuming decay to be a function of base-level activation. Higher activation at the time of practice led to faster forgetting, such as when practice is massed together, and slower forgetting when practice was spaced. Their model accounted for many aspects of practice and spacing. Lindsey et al.^39^ introduced a model of spacing and forgetting and demonstrated its use in an applied setting. Finally, recent work by Walsh et al.^35^ improved upon prior models of spacing by accounting for accelerated relearning after forgetting. Their model compared favorably with alternative models. The success of these models at predicting recall, and their sensitivity to individual item differences, suggests that they can be used to schedule efficient practice.

Although time efficiency is critically relevant for students, it is only occasionally discussed in the context of scheduling practice (e.g., Metcalf and Kornell^16^). It has rarely been included in calculations of optimal practice scheduling according to a model. For instance, Khajah et al.^40^ simulated the effects of using difficulty thresholds to decide practice and found that practicing items close to being forgotten (probability of recall = 0.40) resulted in superior memory performance. This recommendation is similar to Bjork’s^41^ argument that items should be practiced when they are about to be forgotten. However, if a students’ time is a valuable resource, such recommendations by Bjork and Khajah et al. are contradicted by research showing that information is recalled substantially faster at higher probabilities^20,42^. This aforementioned recall probability–latency relationship implies that while a policy such as *p* = 0.40 may improve memory it may be inefficient relative to a policy with a higher probability decision rule (e.g., *p* = 0.80). In short, we agree with the policy of guiding practice based on a predicted probability threshold, but we believe that the time cost of practice at these probabilities needs to be considered when determining an optimal policy.

In sum, conventional spacing schedules can be suboptimal relative to an adaptive scheduling algorithm due to variable item difficulty, the slowing of forgetting as practice accumulates, and the interaction between difficulty and RT. RT is especially relevant if total study time is constrained, but trial duration is not. We also highlighted the possibility of an optimal amount of difficulty to maximize learning, based on arguments by Hebb^43^, Bjork^41^, and Pavlik and Anderson^18^. Tracking probability of retrieval (our operationalization of difficulty) throughout learning was also shown to be achievable, both with our methods and given the existence of several accurate models of memory^38,40,44,45^. The following simulations combine these ideas by evaluating how scheduling according to a model and difficulty thresholds compared to conventional practices schedules and heuristics. An important aspect of our approach was to also simulate RT, which led to different conclusions than prior research. Finally, we tested the simulation predictions with an experiment that compared the relative benefits of valuing efficiency, discrepancy reduction (focusing on more difficult trials), or a traditional nonadaptive approach.

## Results

Simulated students practiced according to various schedules of practice. Correctness probability and RTs were estimated by models with parameters estimated by fitting a dataset (Experiment 1) in which participants practiced Japanese–English word pairs across two sessions wherein spacing, repetitions, and retention interval were manipulated (see methods). See Supplementary Materials for Experiment 1 results.

Each simulated student completed a practice session and a test 3 days later. The primary constraints were (A) the *total* duration of the practice session (22 min, see “Methods”) and (B) that feedback was provided when a retrieval attempt was unsuccessful (which lasted 4 s). We show how conventional practice schedules (e.g., uniform spaced schedule) under conditions of fixed and variable trial durations fare as well as heuristic methods^15^. Finally, we show the effects of scheduling practice based on different OETs that schedule practice according to the probability of correctness.

In all OET conditions, a simulated student practiced whichever item the model estimated was closest to (but less than) the recall probability threshold. If all items were above the threshold, the item closest to the threshold was practiced. The process would repeat until the time ran out.

The primary goals of the simulations were to (a) recreate typical patterns of results found in prior work and (b) evaluate the effects of using a model to schedule practice at different OETs that may balance the benefit of spacing and practice with the time costs associated with higher item difficulties.

### Simulation results

**Fig. 2:**
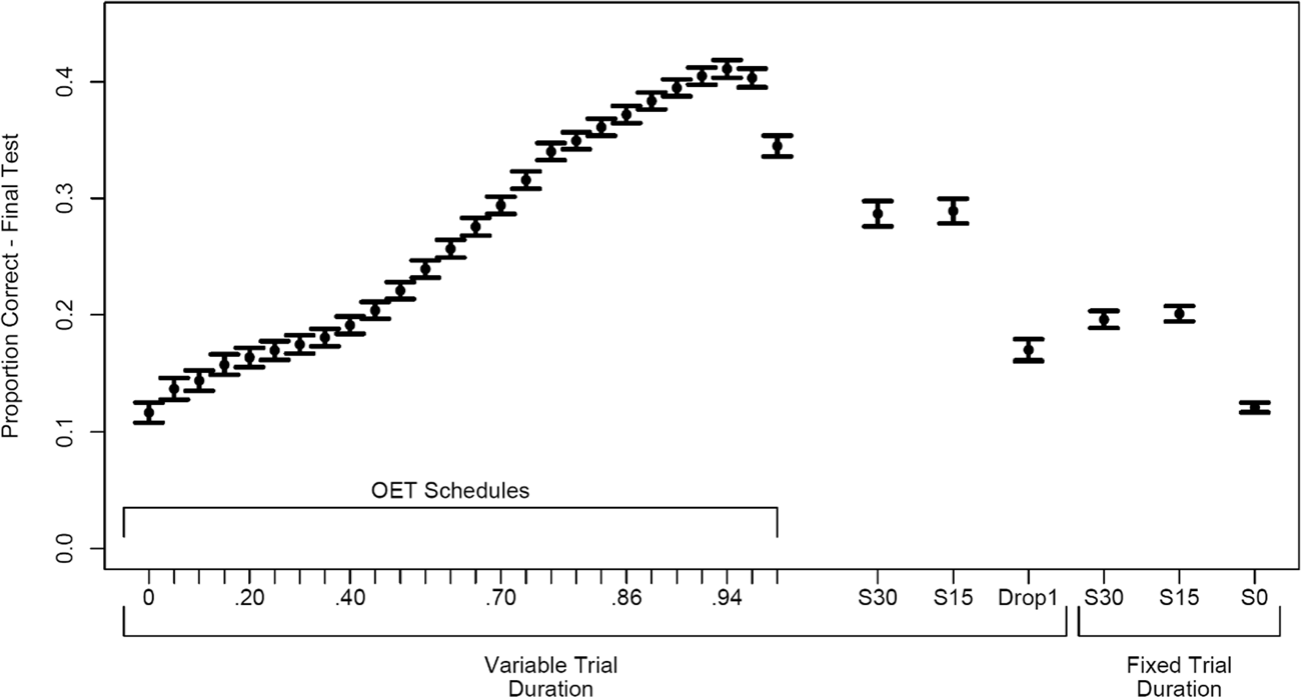
Simulation results for adaptive and conventional conditions. Simulated final test performance after a 3-day delay. There were 200 simulated students per condition. Schedules on the left denote the probability threshold (OETs) for different simulated conditions. 0, 15, and 30 denote spacing in terms of trials (0 being massed). Text on bottom axis shows which simulations fixed trial duration or allowed them to be determined by the latency model (i.e., correct trials being faster than incorrect). Drop1 denotes a heuristic in which items were dropped from practice after successful retrieval, until all items were retrieved and the process restarted. Error bars denote +/−1 SEM.

**Fig. 3:**
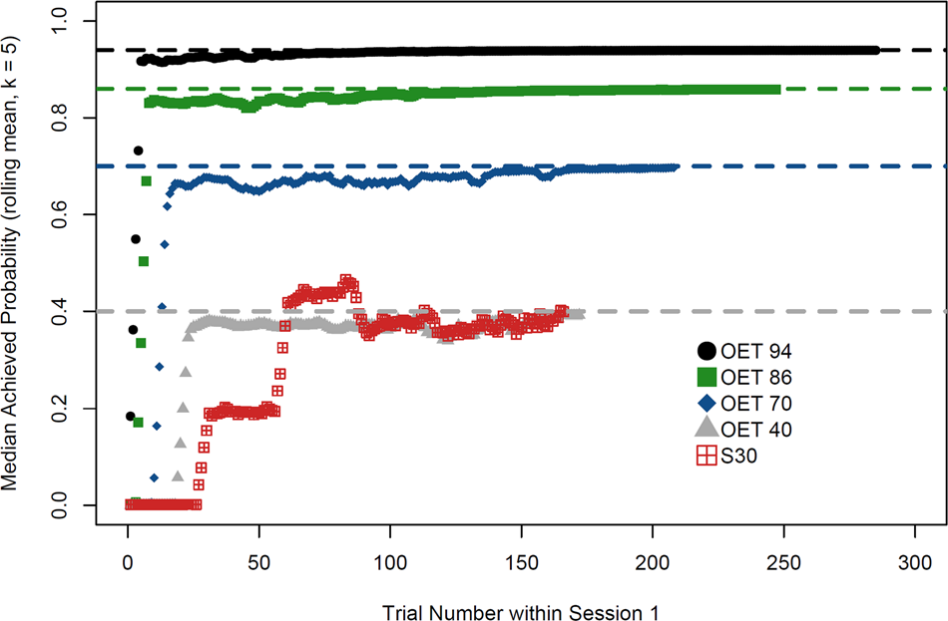
Predicted achieved probability for several conditions. Rolling recall probability (window = 5 trials) of practiced items across 5 conditions. Note that an OET does not dictate the actual probability of recall for practiced items but is a threshold determining what is practiced (closest item underneath threshold). All conditions had the same total time to complete as many trials as possible. Rolling mean computed up to the number of trials *N* completed by 95% of simulated students in each condition. Gray, blue, green, and black dashed lines denote the respective OET thresholds.

Figure 2 depicts many of the critical simulation results. All spaced conditions were superior to the massed condition. Conditions with more spacing also provided a larger benefit than those with less spacing. These results are broadly consistent with what is found in studies comparing conventional spaced and massed conditions^2^. The conventional schedules in which trial duration was determined by the latency model were better than if trial duration was fixed; more trials were possible, especially if simulated students were successful more often. As a result, simulated student skill was highly correlated with the number of trials experienced in conventional schedules without fixed trial durations (see Fig. 6). The best performing conditions were those that adaptively selected which item to practice on each trial according to model predictions and an OET. The best OET in this simulation was 0.94. It is important to note that this does not mean items were always practiced at this probability, especially early in practice (see Fig. 3).

The benefit of this approach was apparent in many of the OET conditions. OETs of ≥0.8 provided 39–55% better memory at final test than the next best conventional schedule. This result indicates that scheduling according to item difficulty and prior practice history while considering efficiency provides superior performance, within the same timeframe, as conventional schedules. Note the achieved probability of the conventional schedule in Fig. 3. On average, conventional schedule probabilities remained low because item difficulty and simulated student ability played no role in deciding what should be practiced next. As a result, items were frequently too difficult. The optimal zone of difficulty we found supports prior suggestions that some difficulty is beneficial^41,43^, with the practical benefit that our method quantified the amount of difficulty, which can in turn be leveraged in combination with a model. Interestingly, the optimal zone that we found indicates imposing less difficulty than previously suggested^40,41^. This is because our methodology considered the time costs of difficulty.

**Fig. 4:**
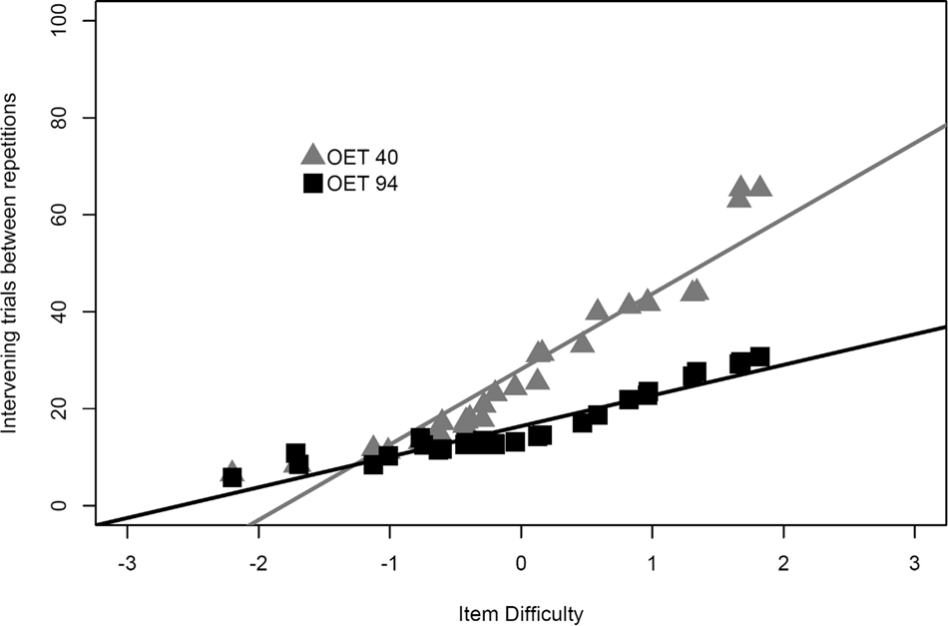
Relation between item difficulty and spacing depends on OET condition. The relation between item difficulty and achieved spacing in OET 40 and OET 94 conditions. In both conditions, easier items (positive intercepts) have wider spacing, while harder items (negative intercepts) have narrower spacing. The increased spacing in OET 40 leads to worse performance. Lines depict a linear regression fit.

**Fig. 5:**
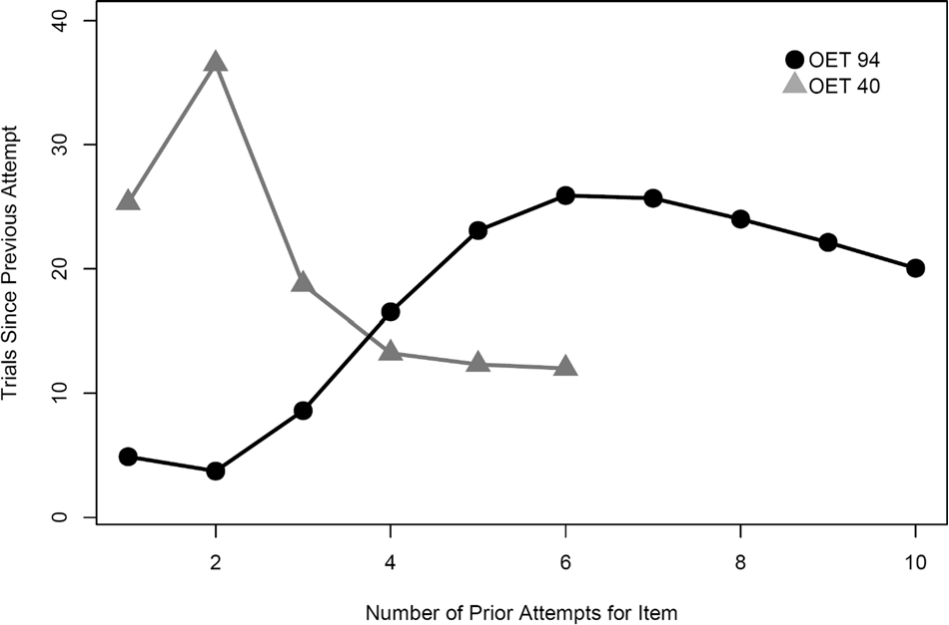
Spacing interval may expand or contract depending on OET condition. The average number of intervening trials since the last attempt as a function of the number of prior attempts in OET 40 and OET 94. In OET 94, as practice accumulates for an item, spacing interval tended to increase. The opposite was true for OET 40, spacing intervals contracted. Each condition plotted to the number of attempts that at least 95% of simulated students attained in the condition (6 and 10 for OET 40 and OET 94, respectively).

Inspection of item-level model behavior provides more examples of the adaptivity of the efficiency-based approach (model+OET). Figure 4 depicts the strong relationship between item difficulty on the *x* axis (item intercept) and average spacing and how it differed between high and low OET simulations. Easier items (higher intercept) experienced wider spacing intervals. This supports findings by Metzel-Baddeley and Baddeley^46^ who showed that applying narrower spacing to harder items benefited memory. Figure 5 show that, as practice progressed, spacing for individual items also widened. This adaptivity means that difficulty adjusted as items were learned, due to the item probability of recall keeping it above the OET (and thus out of contention for practice) for increasingly longer time intervals. Note the positive trend in Fig. 5, which indicates that an expanding schedule can naturally develop within the adaptive scheduling condition. Given the reasonable assumption that items vary in difficulty, non-adaptively conventional schedules are likely to provide practice schedules that are too easy for some items and too difficult for others (and thus inefficient).

**Fig. 6:**
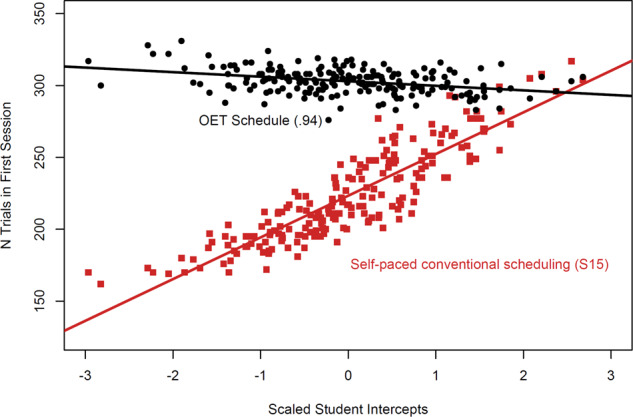
Relation between simulated student intercept and completed trials. The relationship between simulated student intercept and the number of practice trials in two self-paced conditions, an optimal threshold condition (OET 94), and a conventional spaced practice schedule (self-paced with average spacing of 15 trials). Lines indicate linear regression fits. The strong interaction highlights a consequence of the lack of adaptivity in the conventional schedule that leads to better simulated students receiving more practice trials. In contrast, adaptive scheduling led to similar numbers of practice opportunities across various simulated student intercepts.

Simulated student-level skill also influenced the behavior of the adaptive model behavior. In the conventional schedule in which trial duration could vary (and thus the number of trials), the skill of the simulated student (here as the intercept) was strongly correlated with the number of trials (see Fig. 6). Thus “better” simulated students got more opportunities to practice. In contrast, in the adaptive conditions, there was no correlation between simulated student intercept and the number of trials they experienced. The adaptivity of the model meant that difficulty was kept consistent across ability levels and item difficulties. Lower-ability simulated students would receive relatively more practice on easier items than higher-ability simulated students. In other words, the adaptive model+OET is fairer, on top of leading to superior recall.

### Experimental results

This following experiment (Experiment 2 in “Methods,” the parameterization dataset was Experiment 1) was intended to test several related predictions of the simulation. First, we expected high OETs to lead to better memory retention than both conventional schedules and low OETs. However, we also expected OETs beyond the optimal value (e.g., OET = 0.98) to lead to worse performance on a delayed test due to shorter spacing and overpractice of a subset of items. We had participants practice in one of the six conditions. Five were adaptive (OETs = 0.40, 0.70, 0.86, 0.94, 0.98) and were chosen to test the hypothesis implied by the simulation that a difficulty threshold of 0.94 was optimal, with additional conditions 0.40, 0.70, 0.86, and 0.98 to facilitate estimating the shape of the optimality curve. The nonadaptive condition was intended as an additional control (referred to as S15) and repeated items every 15 trials on average. Trial duration was self-paced in all six conditions, except that feedback after incorrect answers lasted 4 s. OET 40 was included to test an alternate theory in which practice is optimal when directed toward more difficult items^33^.

**Fig. 7:**
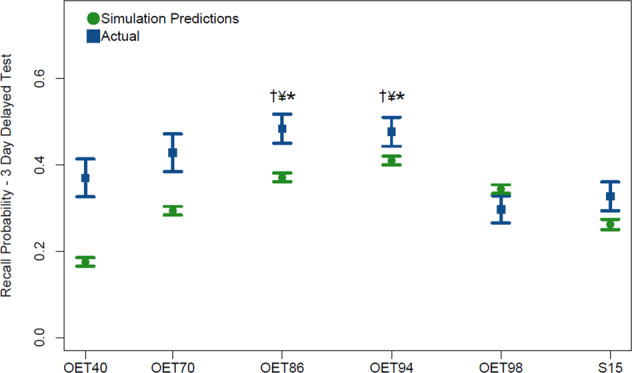
Experimental results testing simulation predictions indicate benefit of high OETs. Experiment 2 results with overlaid original simulation predictions. The symbols †, ¥, and * denote the condition being significantly different than OET 40, OET 98, and S15, respectively. Error bars represent +/−1 SEM.

As can be seen on Fig. 7, final memory test performance in the OET conditions followed a skewed inverted-U shaped pattern as predicted in the simulation. A one-way non-parametric analysis of variance (Kruskal–Wallis *H* test) indicated a significant difference across conditions in average performance on the final test, *H*(4) = 16.5, *p* < 0.01. Post hoc comparisons indicated this difference was driven by the OET 86 and 94 conditions having significantly higher recall performance than the OET 40 (*p*s 0.017 and 0.023, respectively), OET 98 (*p*s < 0.011), or S15 conditions (*p*s < 0.01). There was not a significant difference between OETs 94, 86, and 70, *p*s > 0.17. OET 70 was not significantly different from other conditions, *p*s > 0.07. OET 86 was numerically higher than OET 94 (*M*s 0.48 and 0.47, respectively), and highest overall, but was not significantly different than OET 94, *p* = 0.64. We also compared performance across conditions with a mixed-effects model with random intercepts for items and participants. This analysis indicated the same pattern of statistically significant differences. Including condition as a factor significantly improved the model relative to a model with only random effects, ▫^2^ = 18.72, *p* = 0.002. Final test performance of both OET86 and OET94 was significantly higher than OET 40, OET 98, and S15, *Z*s > 2.83, *p*s < 0.005.

In addition to predicting an advantage of high OETs over lower OETs and nonadaptive schedules, the simulation predicted a skewed inverted U relationship between OET and memory retention at final test. As a basic test of this prediction, we compared the fit of a linear relationship between OET condition value (as a numeric predictor) and average final test performance of each participant to a model with an additional quadratic term. A likelihood ratio test indicated that the model with an additional quadratic term was a significantly better fit (▫^2^ = 23.47, *p* < 0.001) and importantly predicted that performance would eventually decrease as the OET approached 1 (maximally easy). In other words, some difficulty is desirable. The model used to schedule practice fit the data well, McFadden’s pseudo-*R*^2^ = 0.37. McFadden’s scores from 0.2 to 0.4 are considered evidence of a good fit^47^. There was a strong correlation between predicted and actual average performance on the final test, *r* = 0.775, *p* < 0.001. An analysis of the sensitivity and specificity of the model also indicated a good fit, AUC = 0.871, 95% confidence interval = 0.868–0.874.

The OET 94 condition led to approximately 40% higher memory retention than conventional scheduling (S15), as well as the OET 40 condition, which served as a test for a different approach in which higher difficulty items are practiced more often (e.g., a discrepancy reduction approach). The simulation was reasonably successful at predicting the relative ordering and differences among conditions but underestimated performance overall (e.g., experimental OET 94 was ~0.47 but was closer to 0.4 in the simulation). This discrepancy was more pronounced in conditions with higher per-trial difficulty, such as OET 40. This discrepancy could be due to a larger gain from failures or a larger spacing effect than estimated from our model and the previous dataset.

## Discussion

The present research used a computational model to simulate the outcomes of 6 conventional and 22 model-scheduled practice conditions that used different hypothetical OETs. OET 94 was predicted to provide the best recall performance at a final test for learning Japanese–English vocabulary, with several other high OETs providing comparable results. An experiment confirmed that high OETs (0.86 and 0.94) conferred significantly better memory retention at a final test than conventional scheduling and were also superior to adaptive scheduling that focused practice on more difficult items (OET40) or easier items (OET 98). We conclude that, given a correct OET, model-scheduled practice can allow selection of items for practice in a way that is optimal for learning. Our results indicate that practice time is likely to be misallocated in all practice schedules that are insensitive to participants’ practice history, item difficulties, and time costs.

The OET methodology offers several benefits. For one, easier items will go beyond the decision rule threshold sooner and (temporarily) be excluded from practice. Other harder items will then be more likely to be practiced. The OET method also includes a natural mechanism for the critical issue of when to introduce new items—when all other items are currently above the OET. As practice accumulates, inter-trial spacing increases between repetitions in OET-guided scheduling conditions. Thus model-based scheduling naturally leads to an expanding schedule. The critical difference that distinguishes the conventional expanding schedule from the model-based schedule is that in the conventional schedule the exact interval between repetitions is identical for all items regardless of difficulty. Here our expanding schedules were adaptive based on the dynamic model predictions at the item level.

One rebuttal to the proposed model-based approach may be that it requires too much information to be useful (i.e., model parameters). However, it is quite rare for content to be taught for the first time to a student. There is frequently prior data from which to estimate model parameters and intercepts of the items themselves. The difficulty is in whether the dataset contains the necessary conditions from which to fit the model (e.g., variety in spacings and repetitions) or whether it is biased in some way that precludes meaningful model fitting. These potential issues can be overcome by embedding experimental conditions within educational systems^45^ or running controlled experiments during the educational system design process. Our experiment demonstrated that adaptively scheduling practice using model predictions and difficulty thresholds (OETs) is both possible and can benefit memory. While more data across a wider variety of spacing intervals and learning contexts will be helpful to strengthen these arguments, we believe these methods will generalize to tasks where failure has larger time costs than success and where forgetting is a substantial effect.

**Fig. 8:**
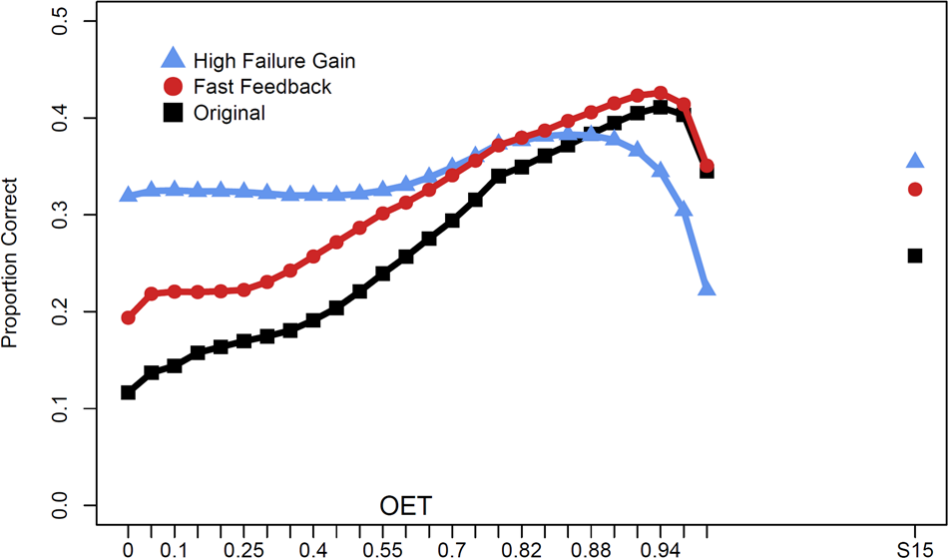
Results of simulations with alternate assumptions indicate that optimal difficulty threshold may vary. The original simulation (in black) as well as two additional simulations in which the efficiency of failures is increased. An alternative simulation in red shows performance if feedback time cost for failures was set to 0 s (instead of 4). Another simulation in blue shows performance if failures provided 25% more learning gains than successes (but time cost was same as original). Optimal threshold for paired associate learning remains high for all of them, but the relative benefits over lower OETs is clearly influenced by gains from failures and time cost. S15 (repetitions every 15 trials, trial duration not fixed) was also simulated under the three sets of assumptions and serves as a point of comparison.

In the present study, we focused on word pair learning as a simplifying assumption. Such materials are common in learning research and allow easier interpretation of results due to the independence among the items. Of course, learners do need to learn vocabulary and sets of facts, but those materials differ in ways that justify testing the OET approach with other materials. For one, the speed and learning gains of successes and necessity of feedback with paired associates means that a high OET will tend to perform best. But with materials in which failures provide larger gains, or are faster, lower OETs may be preferable (see Fig. 8). Additionally, another important property of other educational materials is that they contain educationally relevant interrelations. Complete models of memory will need to account for such interrelations and model possible learning transfer among related concepts. An important extension of the present research will be to account for such transfer to improve adaptive scheduling of educationally relevant learning materials and the possible changes in scheduling and OET selection.

A further improvement could be to adjust model parameters based on model prediction errors^48^. Relatedly, recording students’ preferred study targets may be helpful for improving the model, and there is evidence that their metacognitive judgments can positively improve practice selection^17^. However, this limitation to the adaptivity of the present model does not detract from the general conclusion that scheduling practice based on the most *efficient* threshold is superior to any conventional schedule, just that the model to estimate probability could be adjusted to adapt to its own performance.

Readers may have inferred that sometimes items would be practiced far from the intended OET, typically early in practice. The efficiency of trials early in practice (or for items that are especially difficult) could be improved by adaptively manipulating the strength of retrieval cues. For instance, Fiechter and Benjamin^49^ demonstrated how adaptively changing cue strength can benefit memory (e.g., cue—Targ__ instead of cue—____). Adaptive cueing could be integrated into the model+OET framework by estimating the probability of recall for each item with different cue strengths. This would change the decision rule from selecting among one probability per item to each item having multiple probabilities due to varying cue strengths. A newly introduced item would have strong cues (e.g., cue—Targ__), gradually weakening until “cue—____” was the only cue under the OET for that item.

Features of the adaptive learning context (e.g., variable numbers of trials and lower difficulty) may meaningfully influence performance and need to be studied further. For instance, in high OET conditions, there is likely to be less interference, because new items are gradually introduced, reducing the average difficulty of intervening practice. In other words, at any given moment most items that could interfere with retrieval tended to be more well learned as a function of OET, which was not necessarily the case in traditional practice schedules or our initial parameterization experiment. The model we used did not account for this varying interference during practice and may explain why our simulation underestimated the benefit of high OETs, similar to results of Pavlik and Anderson^18^, which showed better learning of fixed schedule items under conditions of reduced difficulty of the surrounding adaptively scheduled practice.

We have emphasized throughout that we are not arguing for the superiority or the use of a particular model of memory. There are several that have been shown to accurately model spacing and practice effects in various learning contexts^34,35,38,50^. In our view, accurately estimating recall probability is not the primary issue. Rather, our focus has been on how these computational models allow scheduling to be sensitive to individual differences and *efficiency*. We focused on using the model to estimate recall probability, given a history of practice, and on using those estimates to practice items closest to a target threshold of optimal difficulty. Our argument differed from prior research in how we believe an optimal threshold should be chosen and how the efficacy of practice should be evaluated. Considering time cost in our simulations (making them sensitive to efficiency of practice) led to different conclusions than prior recommendations—the relationship between latency and recall probability, coupled with the large time costs of being incorrect, meant that practicing paired associates at an OET of 0.40 was less efficient than higher OETs. Our experimental data supported our arguments—practicing paired associates near an optimal high OETs caused significantly higher memory retention than alternative OETs as well as better than a conventional spaced practice condition.

However, a high OET may not always be preferable. In fact, it will always be the case that, if there is any spacing effect or advantage to difficulty, as the OET approaches 1 (maximally easy), efficiency will decline. An OET of 1 can only be efficient if learning is optimal with perfect correctness (i.e., if Skinner was correct). A higher OET will be best when gains from successes are equal to or greater than failures while also being faster. This is true for many learning materials but not necessarily all of them. Situations in which failures provide more learning gains or are faster will result in lower recommended OETs.

The critical takeaway of the present work is that, if an accurate computational model is used, adaptively scheduling practice with model predictions according to an *optimal efficiency threshold* will confer superior memory relative to conventional practice schedules. Although the exact value of the OET will vary across learning materials and types of feedback, practice efficiency will be relevant unless the learning gains and time costs of successes and failures are equal. Our simulation and results demonstrate how important this consideration can be for scheduling practice. The benefit of our approach relative to conventional spacing was clear (Cohen’s *d* = 0.64) and comparable in magnitude to that of the spacing effect itself^51^ (Cohen’s *d* = 0.42).

Finally, shifting the focus away from fixed schedules and trial durations and toward an adaptive framework offers new exciting avenues of research, such as modifying the component parts of the adaptive framework (models for predicting correctness and RT, estimating OETs) to accommodate learning more complex materials that contain inter-item relations.

## Methods

### Experiment 1 methods (model parameterization)

#### Materials

Learning materials were Japanese–English word pairs. Pairs were chosen such that the target English words contained four letters and had average familiarity and imageability according to the MRC Psycholinguistic Database. Each participant studied 48 Japanese–English word pairs, divided equally among the conditions described below.

#### Participants

The experiment was approved by the institutional review board of the University of Memphis. Informed consent from obtained from all participants before the task began. One hundred and thirty-two participants were recruited via Amazon Mechanical Turk. Sixty-six Participants were female, and 59% were aged between 18 and 34 years. Our sample size left us highly powered to detect within-participant effects of practice and spacing (e.g., >90% chance to detect an effect of *d* = 0.28) and the likely large between-participant effect of retention interval on final test recall. There were 43, 45, and 44 participants in the 2-min, 1-day, and 3-day retention interval conditions, respectively. These retention intervals were chosen to measure the curvature of the forgetting function over the intervals relevant for the planned simulation, for which we expected rapid initial forgetting (hence the inclusion of the 2-min condition).

#### Method

Participant data collection was completed using the MoFacTS system, which is a software designed to allow practice scheduling according to a model as well as more traditional approaches^52^. Participants completed two practice sessions. In the first session, the number of learning trials per item (2, 4, 8) was manipulated within participants. The amount of spacing between these trials was also manipulated within participants; the number of intervening trials could be very narrow (0 or 1 intervening trials), narrow (approximately 4 intervening trials), wide (approximately 8 intervening trials), or very wide (approximately 13 intervening trials). To reduce the chance that participants would recognize particular schedule patterns, the exact positions of item repetitions within a sequence were randomly jittered (e.g., +/−1 position from original assignment). The delay between the first and second sessions was manipulated between participants and was 2 min, 1 day, or 3 days. In the second session, participants were tested three times with feedback on each item. The items in the second session were presented in blocks, randomized within each block. In other words, items in the second session were not tested again until all other items had been tested.

There were two types of trials possible in both experiments (and the simulations). The first trial for all items was a study trial, in which the intact Japanese–English word pair was presented for 7 s. Subsequently, all trials for each item were test trials, in which participants were presented with the Japanese word as a cue and asked to type in the English target word (e.g., “awa—____”). Participants had 7 s to type in an answer and press enter to continue. If they were correct, a brief “correct!” message would appear for 500 ms. Then there would be a 500-ms delay before the next trial began. If they were incorrect (or did not provide an answer), the intact word pair would be presented for 4 s along with a message telling them they were incorrect or that time had expired. To ensure that participants were not cut off while typing their answer (e.g., if they began typing after 6.9 s had elapsed), the timeout timer for the trial reset once the participant began typing. This accommodation was rarely necessary (approximately 4.7% of trials, across both sessions).

#### Simulation model fitting

For the simulations, we fit a logistic regression model (see Eq. (2)) to predict recall probability, inspired by PPE^35^ and Base4^50^.

2 $$y = \beta _1a^{ - d_{\rm{s}}}N_{\rm{s}}\,S^c + \beta _2a^{ - d_{\rm{f}}}N_{\rm{f}}\,S^c.$$

There are two additive components, meant to allow differential contributions of successes and failures. Parameter *a* represents the time since first practice, with *d* representing a decay parameter (separate for successes and failures). *N* represents counts of prior practice (separate for successes and failures). Parameter *S* is average spacing in between practice attempts, with curvature parameter *c*. See Supplementary Materials for more details. The model was fit with intercepts for participants and items. There are many candidate models that would probably account for the general patterns found in the present data. However, we want to emphasize that our motivation is not to find the best computational model of memory but rather to investigate how to *use* such models. We believe the general conclusions of the results below would extend to other computational memory models. A model for RT was also fit. The RT model allowed the total number of trials within a fixed duration to vary according to correctness, given that correct answers would be faster than incorrect answers (see the Supplementary Materials for a simulation using a simpler RT model that provided similar results). The RT model was as follows:

3 $${\rm{RT}} = ce^{ - a} + fc,$$

with the two free parameters *c* and *fc* estimated via maximum likelihood. Parameter *a* was the log odds prediction from the correctness model described above^23^. In other words, the RT model sought to translate the correctness model predictions into RT. This model was solely intended to track RT for correct answers (RT for incorrect answers is typically estimated with a constant^18^).

#### Simulation methods

Each run of a simulation represented 2 sessions of a single student studying 30 word pairs for 22 min, followed by a test 3 days later. The total duration and number of items was chosen to match the duration and practice characteristics of typical paradigms in which participants study approximately 30 items, practicing them 4 times each, with 7 s trial durations for initial study trials, 11 s for subsequent tests with feedback (7 + 4 s), 0.5 s inter-stimulus interval (ISI), for a total of 1320 s (22 min). We believe simulating a scenario that is common to the literature on this topic makes it easier for researchers to compare our findings and conclusions to prior literature. Two hundred students were simulated for each condition. Item and student intercepts were sampled from their respective distributions estimated from fitting the model on the experimental dataset. As a concrete example, to compute the probability of success on a given trial for each item, the computational model would predict recall probability with the (simulated) history of practice for each item for the student as well as the intercept for that particular item and student. The correctness of a trial was determined probabilistically using the prediction from the model (e.g., if the model predicted 68% probability of recall, the trial was correct with 68% probability).

**Fig. 9:**
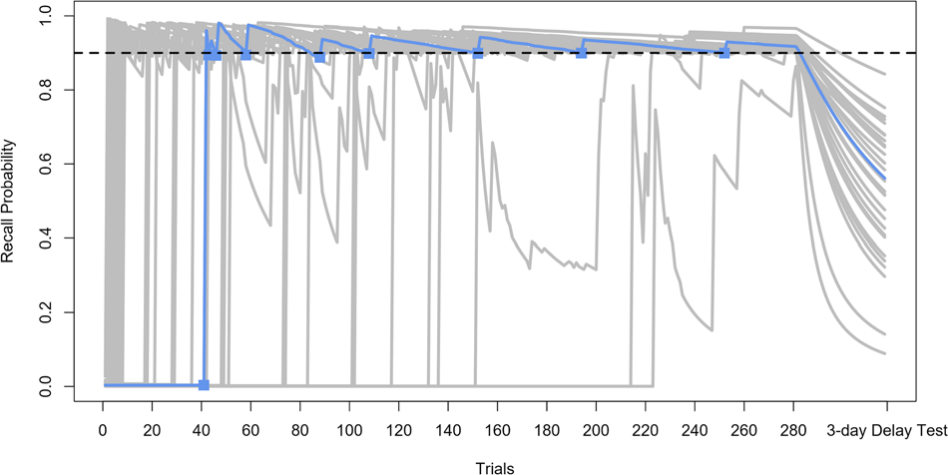
Estimated probability of all items for one simulated student. A plot of the estimated probability of all items during optimized practice for one simulated student (at OET = 0.90). The blue line indicates the probability of one item across the sessions. Squares indicate when that item was chosen to be practiced. Gray lines represent other items (squares indicating when other items were practiced were omitted to avoid clutter). The black dashed line denotes the OET.

Simulated students that were in “optimal” conditions practiced whichever item was closest (but less than) the target OET for their condition (e.g., 90%). For instance, if an item was practiced at 89%, its post-practice estimated probability would go beyond the optimal value (90%), and it could not be practiced again until it decayed to <90%. If all items had a predicted recall probability greater than the OET, the item with the closest probability was chosen. Figure 9 shows a time course of item probabilities for a student practicing in the 90% optimal condition, with one item highlighted to show the consequences of scheduling practice according to the computational model and the 0.90 OET.

#### Optimal probability (OET) conditions

These conditions differed according to their target probability threshold at which to practice (or the decision rule). There were 22 optimal probability conditions depicted here, in intervals of 0.05 from 0.2 to 0.8 and intervals of 0.02 from 0.8 onward to 0.98. We simulated across the range to illustrate the efficiency curve; the particular intervals were simply intended to help the reader gauge the general pattern. For example, a student in the 0.80 condition would practice whichever item was closest to (but less than) 0.8 probability. Items above the optimal value were not practiced until they decayed below the threshold unless all items were above the threshold, in which case whichever item was closest to 0.8 would be practiced.

#### Conventional conditions

Given that our focus is on practice of word pairs or other similarly independent facts, we simulated schedules common to that literature. Schedules could be simulated either with a fixed trial duration (common to the spacing literature) or a variable trial duration in which trial duration was determined by the latency model. One adaptive heuristic schedule was included (Drop-1), in which an item was dropped from practice after being recalled (if all items were answered correctly before the time elapsed, the process restarted). Thus, in fixed trial duration conditions the number of trials was also fixed, while in variable duration conditions the number of trials could vary. If a student was successful more often, and thus faster, they could complete more trials within the maximum allotted time.

The critical comparison was among the conventional variable duration spacing conditions, Drop-1, and the OET conditions, because in these three types of conditions successes are faster and may lead to additional practice attempts. The question is whether employing an OET (e.g., the rule “practice whichever item is nearest but <0.90 recall probability”) improves the efficiency of practice above and beyond conventional spacing schedules that do not use a model.

#### Simulating latency

Although computational models do not necessarily differentiate between successes and failures (counts of all attempts are frequently used instead), outcomes still have important consequences for trial duration. Most importantly, failure trials may require feedback, whereas successful trials may not. Given that failures (with feedback) and successes (with or without feedback) can provide the same memorial benefit^53^, successful trials can be more efficient per second. In simulated “optimal” conditions, trial duration was decided based on success and failure and thus also influenced the total number of completed trials for a student within the allotted time.

The duration for the first trial for each simulated student was set to 7 s, to represent an initial study trial. Subsequently, if the condition allowed the simulated student to proceed at their own pace, the duration of correct trials was estimated with the latency model described above plus 500 ms to simulate the correctness feedback. Incorrect trial durations were set to be the median trial duration for incorrect trials found in the experimental data plus 4 s feedback (8.98 s total). In simulated conditions with fixed trial durations (common in many experimental designs), the duration was fixed at 11 s (7 s to recall plus 4 s feedback). There was a 1-s ISI between trials in all conditions. The values chosen for trial duration and ISI are typical for spacing experiments^45^.

### Experiment 2 methods

#### Materials

Materials were Japanese–English word pairs used in the parameterization dataset. Each participant was assigned a randomly selected subset of 30 word pairs to practice.

#### Participants

The experiment was approved by the institutional review board of the University of Memphis. Informed consent from obtained from all participants before the task began. In Experiment 2, 322 participants recruited from Amazon Mechanical Turk (Mturk) via TurkPrime^54^ completed the task. Data from 25 participants were excluded from analysis for reporting that they had at least moderate ability to read the Japanese language. Six participants were excluded for showing no evidence of completing the task (e.g., timing out on all trials). Participants were excluded at similar rates across the conditions. We aimed for approximately 50 participants for each condition. The one exception was OET 98, in which we aimed to collect fewer participants (20) because simulations indicated that participants would overpractice a small set of the items and thus there would be very little variance. In other words, we were very confident the condition would be suboptimal, and we collected data in it solely to concretely demonstrate the inefficiency of extremely high OETs. Ultimately, the *N*s per condition were 59, 53, 57, 50, 56, and 16 for S15 and OETs 40, 70, 86, 94, and 98, respectively. The number of participants was chosen based on effect sizes observed in past research on adaptive practice scheduling^18,33,55^. These prior findings indicated the potential for a medium-to-large effect size (Cohen’s *d* of 0.56 to >1). We powered our experiment to detect an effect size of *d* = 0.55. Using the BUCSS statistical package^56^, which corrects for publication bias and sample uncertainty, we estimated we had approximately 89% probability to detect a significant difference among conditions.

#### Method

Participant data collection was again completed using the MoFacTS system^52^. All participants completed two sessions. In the first session, after providing informed consent, participants studied 30 word pairs for 22 min. Twenty-two minutes was chosen to approximately match previous research on learning word pairs that typically involves studying about 30 word pairs a few times each^13,28^. Twenty-two minutes also ensured four exposures to each item during the first session given maximum trial durations. The format and duration for initial study trial for items, subsequent test trials, and feedback for correct and incorrect trials was the same as Experiment 1. As before, when a participant began typing an answer, the timer would reset so the trial did not end as they were typing. This accommodation again rarely extended the test portion of the trial beyond 7 s (~3% of trials). After completing the first session, all participants completed a brief survey asking them for demographic information as well as their knowledge of Japanese vocabulary. Participants were informed that their answers would not influence their payment. Data from participants that reported knowledge of Japanese vocabulary prior to the experiment were discarded. The conditions differed in terms of how practice was scheduled during the first session, described below.

Three days later, an email was automatically sent to all participants inviting them to return for a final test. Participants were allowed up to 48 h from when they received the email to complete the final test. During the final test, participants were tested for their memory of all Japanese–English word pairs selected for them during the first session. The order in which word pairs were tested was randomized, and feedback was presented in the same way as in the first session (i.e., a brief “correct” message if correct, and corrective feedback if incorrect).

#### Model

The practice scheduling model was highly similar to what was fit to the parameterization dataset and used to schedule practice in the simulation. The only difference was that, due to not having participant intercepts (due to having not collected data from these new participants yet), the participant intercept was excluded. The previously estimated parameters were fixed (e.g., spacing and decay parameters), and the model was refit with only item intercepts (because we do have previous data for all word pairs from the previous experiment).

#### Adaptive scheduling method

If participants were assigned to an adaptive scheduling condition, they practiced according to 1 of the 5 difficulty thresholds—0.40, 0.70, 0.86, 0.94, or 0.98. This approach proceeded as follows: for each trial, the probability that each item could be recalled was estimated by the model (the difficulty). This estimate was determined by the model using the prior history of practice for that item for that participant (including counts of successes, failure, average spacing, and elapsed time since those attempts), or if there was no prior history just the item intercept was used. Whichever item was closest to (but less than) the difficulty threshold was chosen to be practiced. If all items were above the threshold, the item closest to the threshold was chosen. After each trial, the model re-estimated the recall probabilities for all items, and the process continued until the allotted time elapsed.

#### Conventional scheduling method

If participants were assigned to the conventional scheduling condition, their practice was separated into two consecutive blocks, with 15 items randomly assigned to be practiced within each block. In each block, items were repeated approximately every 15 trials (maximally spaced within block). The exact ordering was slightly jittered to reduce the probability that participants could predict the presentation of upcoming items. We chose this spacing interval due to evidence in initial pilot studies that maximal spacing within one block (i.e., repeating every 30 trials) was exceedingly difficult. Maximal spacing has been shown to be suboptimal in prior research as well^2^, and so this implementation may have served as a stronger control. The implementation of initial study trials, test trials, and feedback (when incorrect) was the same as in the difficulty threshold conditions.

### Reporting summary

Further information on research design is available in the Nature Research Reporting Summary linked to this article.

## Supplementary information

Reporting Summary

Supplementary Materials

## Data Availability

The experimental data are available at https://osf.io/d9rms/ and https://datashop.memphis.edu/.

## Code availability

Simulation script (with embedded instructions) is available at https://osf.io/d9rms/.
