# Supplementary material for: Optimizing practice scheduling requires quantitative tracking of individual item performance
Source: NPJ Sci Learn. 2020 Oct 15;5:15. doi: 10.1038/s41539-020-00074-4 (PMC7567101; doi:10.1038/s41539-020-00074-4)
Supplement: Supplementary file 1 — Reporting Summary [file 41539_2020_74_MOESM1_ESM.pdf]

nature research

Corresponding author(s): Luke G. Eglington

Last updated by author(s): Jun 30, 2020

Reporting Summary

Nature Research wishes to improve the reproducibility of the work that we publish. This form provides structure for consistency and transparency in reporting. For further information on Nature Research policies, see our Editorial Policies and the Editorial Policy Checklist.

Statistics

For all statistical analyses, confirm that the following items are present in the figure legend, table legend, main text, or Methods section.

n/a	Confirmed	
☐	☒	The exact sample size (*n*) for each experimental group/condition, given as a discrete number and unit of measurement
☐	☒	A statement on whether measurements were taken from distinct samples or whether the same sample was measured repeatedly
☐	☒	The statistical test(s) used AND whether they are one- or two-sided<br>*Only common tests should be described solely by name; describe more complex techniques in the Methods section.*
☐	☒	A description of all covariates tested
☐	☒	A description of any assumptions or corrections, such as tests of normality and adjustment for multiple comparisons
☐	☒	A full description of the statistical parameters including central tendency (e.g. means) or other basic estimates (e.g. regression coefficient) AND variation (e.g. standard deviation) or associated estimates of uncertainty (e.g. confidence intervals)
☐	☒	For null hypothesis testing, the test statistic (e.g. *F*, *t*, *r*) with confidence intervals, effect sizes, degrees of freedom and *P* value noted<br>*Give P values as exact values whenever suitable.*
☒	☐	For Bayesian analysis, information on the choice of priors and Markov chain Monte Carlo settings
☒	☐	For hierarchical and complex designs, identification of the appropriate level for tests and full reporting of outcomes
☐	☒	Estimates of effect sizes (e.g. Cohen's *d*, Pearson's *r*), indicating how they were calculated

Our web collection on statistics for biologists contains articles on many of the points above.

Software and code

Policy information about availability of computer code

Data collection	Data collected using the MoFaCTS system. MoFacts code is available here: https://github.com/memphis-iis/mofacts-ies. Simulation code is available at https://osf.io/d9rms/.
Data analysis	Data analyzed using R version 3.5.2.

For manuscripts utilizing custom algorithms or software that are central to the research but not yet described in published literature, software must be made available to editors and reviewers. We strongly encourage code deposition in a community repository (e.g. GitHub). See the Nature Research guidelines for submitting code & software for further information.

Data

Policy information about availability of data

All manuscripts must include a data availability statement. This statement should provide the following information, where applicable:

- Accession codes, unique identifiers, or web links for publicly available datasets
- A list of figures that have associated raw data
- A description of any restrictions on data availability

Data is available at https://osf.io/d9rms/ and https://datashop.memphis.edu/

Field-specific reporting

Please select the one below that is the best fit for your research. If you are not sure, read the appropriate sections before making your selection.

☐ Life sciences ☒ Behavioural & social sciences ☐ Ecological, evolutionary & environmental sciences

For a reference copy of the document with all sections, see nature.com/documents/nr-reporting-summary-flat.pdf

Behavioural & social sciences study design

All studies must disclose on these points even when the disclosure is negative.

Study description	The study was quantitative. Data were collected from human participants. A mathematical model (see supplemental materials for parameter estimates) inspired by prior research on memory decay, spacing, retrieval practice, and cognitive modeling. Model parameters were estimated by fitting to behavioral data (initial experiment). That model was used to simulate participants practicing according to model predictions of practice difficulty and different difficulty thresholds. Subsequent experiment tested simulation predictions. Final experiment tested simulation predictions on human participants.
Research sample	Participants were recruited via Amazon Mechanical Turk.
Sampling strategy	Minimum sample size was determined based on prior literature (see methods). We collected data to have >85% power in our experiments.
Data collection	Data was recorded automatically and stored in a secure server. Participants completed the task remotely and were randomly assigned to conditions. The researcher was not present while participants completed the task.
Timing	Data collection was from August 2019 to May 2020.
Data exclusions	33 participants were excluded for reporting knowledge of Japanese vocabulary (the stimuli in the experiment).<br>6 participants were excluded for timing out on all trials in the first session (e.g., not typing responses at all).
Non-participation	Approximately 75% of participants that completed first session returned and completed the second session.
Randomization	Participants were randomly assigned to conditions.

Reporting for specific materials, systems and methods

We require information from authors about some types of materials, experimental systems and methods used in many studies. Here, indicate whether each material, system or method listed is relevant to your study. If you are not sure if a list item applies to your research, read the appropriate section before selecting a response.

Materials & experimental systems

n/a	Involved in the study	
☒	☐	Antibodies
☒	☐	Eukaryotic cell lines
☒	☐	Palaeontology and archaeology
☒	☐	Animals and other organisms
☐	☒	Human research participants
☒	☐	Clinical data
☒	☐	Dual use research of concern

Methods

n/a	Involved in the study	
☒	☐	ChIP-seq
☒	☐	Flow cytometry
☒	☐	MRI-based neuroimaging

Human research participants

Policy information about studies involving human research participants

Population characteristics	See above.
Recruitment	Participants were paid for their time via Amazon Mechanical Turk. Tasks are posted on the Mechanical Turk website, and so only participants with computers and knowledge of the website could participate.
Ethics oversight	The ethics review board of the University of Memphis.

Note that full information on the approval of the study protocol must also be provided in the manuscript.
