# Supplementary material for: Optimizing practice scheduling requires quantitative tracking of individual item performance
Source: NPJ Sci Learn. 2020 Oct 15;5:15. doi: 10.1038/s41539-020-00074-4 (PMC7567101; doi:10.1038/s41539-020-00074-4)
Supplement: Supplementary file 2 — Supplementary Materials [file 41539_2020_74_MOESM2_ESM.pdf]

Supplementary Materials

Experiment 1 Results

Experiment 1 was designed to provide data from varied schedules of practice and numbers of repetitions to facilitate parameterizing the model and generate simulations.

Results

Typical patterns of practice and spacing were found, as can be seen in Supplementary Figure 1. Importantly, wider spacing resulted in better final test recall than narrower spacing in all retention intervals (two-sided *t*s > 3.1, Cohen's *d*s > .48), replicating the spacing effect. Retention interval had a strong effect on final test memory with longer delays leading to significant forgetting between each retention interval (2 minute, *M* = .424, 95% CI = .366–.482; 1 day, *M* = .258, 95% CI = .196–.319; 3 day, *M* = .149, 95% CI = .099–.198). The dataset replicated classic patterns expected from varying spacing, practice, and retention intervals. For instance, there was a significant interaction between spacing interval and retention interval, Z = 2.64, *p* = .007. This interaction indicated that the benefit of spacing was larger following larger retention intervals. There was also a significant interaction between the number of repetitions and spacing width that indicated the benefit of spacing increased as the number of repetitions increased, Z = 3.60, *p* = .0002. These patterns are typical of spacing studies, and suggested that the dataset was a good candidate to be fit by the computational models and could provide reasonable parameter estimates. The model (described in detail below) fit the average performance of conditions well, R^2 = .95 (see Supplementary Figure 1).

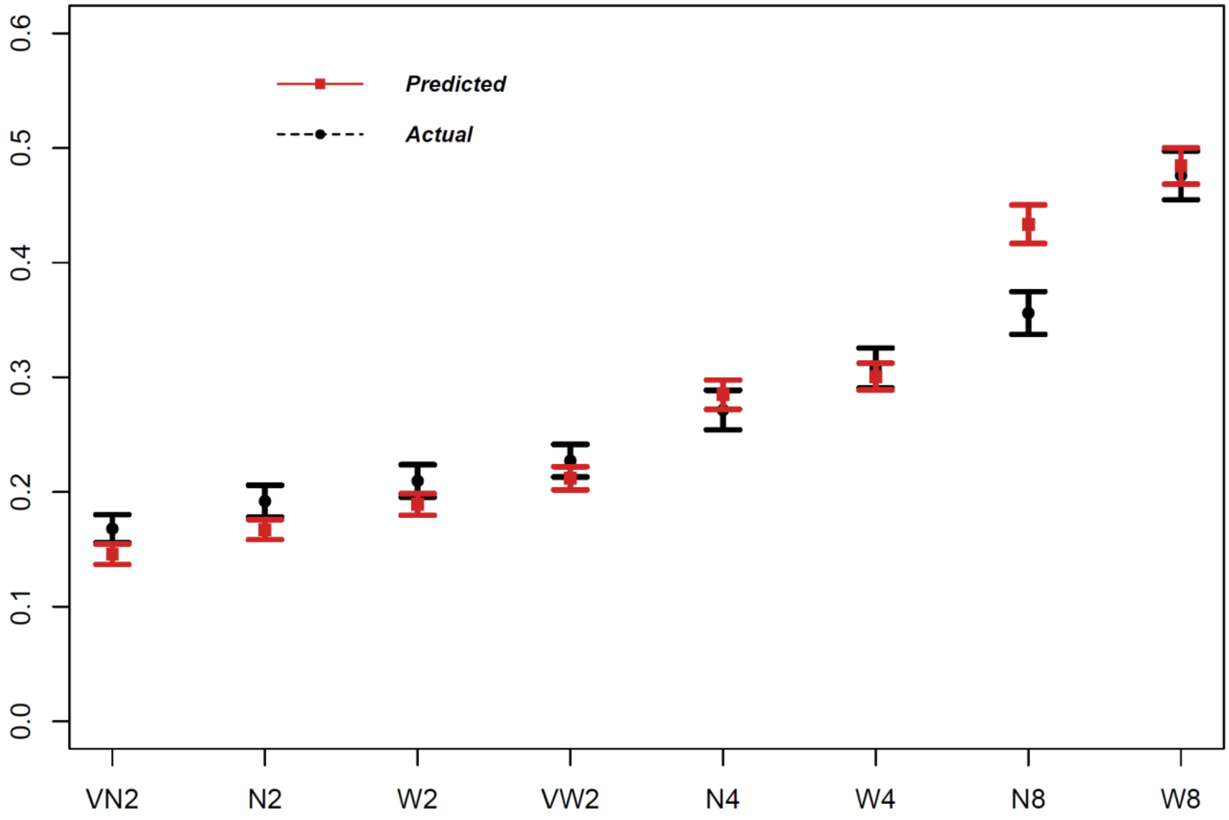

Supplementary Figure 1. Actual means per condition (black) and predicted by model (red) collapsed across all between-participant delays (2 minutes, 1 day, or 3 days). W, N, VN, and VW indicate wide, narrow, very narrow (massed), and very wide spacing, respectively. X-axes label numbers indicate number of trials per item in the first session (e.g., W2 = wide spacing, two trials). Error bars represent +/- 1 SEM.

Model Parameters

Below we describe the equation (1) parameters in more detail.

$$y = \beta_1 a^{-d_s} N_s \, s^{c1} + \beta_2 a^{-d_f} N_f \, s^{c1}, \tag{1}$$

Success and failure trials had separate parameters for decay (*d*) and for the steepness of the learning curve (*L*). The parameters for interference (int), average spacing (*s*), spacing curvature (*c1*), and the effect of the initial spacing (*IS*) were the same for successful and unsuccessful trials.

Ns (counts of successes). *Log*(*Ls* + success count).

Nf (counts of failures). *Log*(*Lf* + failure count).

a (age of item that weighted time spent studying other items differently using an interference parameter). (true.age – interference.age)**int* + interference.age.

Decay parameters. The memory trace or activation of trials was allowed to decay at different rates for successes vs. failure trials.

Interference parameter. This parameter is intended to represent the increased interference that occurs within a session compared with between a session. In other words, we are assuming that the effect of time is less between sessions than within a session. This is achieved by multiplying the amount of elapsed time (in seconds) that is not trial time (and thus is inter-session time) by a scalar.

Spacing parameters. Parameter c moderated the effect of average spacing (*s*). An additional parameter c2 determined the influence of the initial space between the first and second trials for an item.

Learning curve parameters (Ls and Lf). Separate parameters for successes and failures allowed different learning rates (e.g., log(0.1 + N) vs. log(10 + N)).

Supplementary Table 1

Parameter	Success	Failure
Decay (d)	.2365	.1720
Interference (*int*)	.0145	.0145
Spacing (c1)	.0861	.0861
Spacing (c2)	.1137	.1137
Learning Curve	.1301	1
β coefficient	6.26	4.08

The latency model used in the simulation was fit via maximum likelihood to the latencies of correct answers in the parameterization dataset. The values *c* and *fc* were found to be .395 and 3.09 respectively, with the *a* in the model being the logit predicted by the

correctness model described above. For instance, if the correctness model predicted a .95 probability of correctness (logit = 2.95), then the latency model would predict a trial duration of .395*exp(-2.95)+3.09 = 3.11 seconds. For a weaker memory with a lower probability of .5 (logit = 0), the model would predict .395*exp(-0)+3.09 = 3.49 seconds.

Alternate Simulation

Rather than estimate response time with a model, simulated response time for correct answers could instead be assumed to be a fixed value. We set the value for correct answers to be the median response time found on correct answers in the parameterization dataset (2.96 seconds). Incorrect answer trial durations were still set to be the median response time found for incorrect answers in the parameterization dataset (4.83 seconds) plus 4 seconds for feedback. Thus there was still was reduced time cost for correct answers. Supplementary Figure 1 below depicts the results, which are actually quite similar to that shown in the main text. This is partially because the response time model we used is close to asymptote when probability of correctness is greater than .6, and so many of the simulated OET conditions are not greatly affected. If trials took a greater amount of time or were more complicated (e.g., multi-step mathematics problems) results would likely vary more substantially. In either case, the conclusion is clear – if correct answers are faster, practicing according to a model and an OET is preferable to conventional schedules.

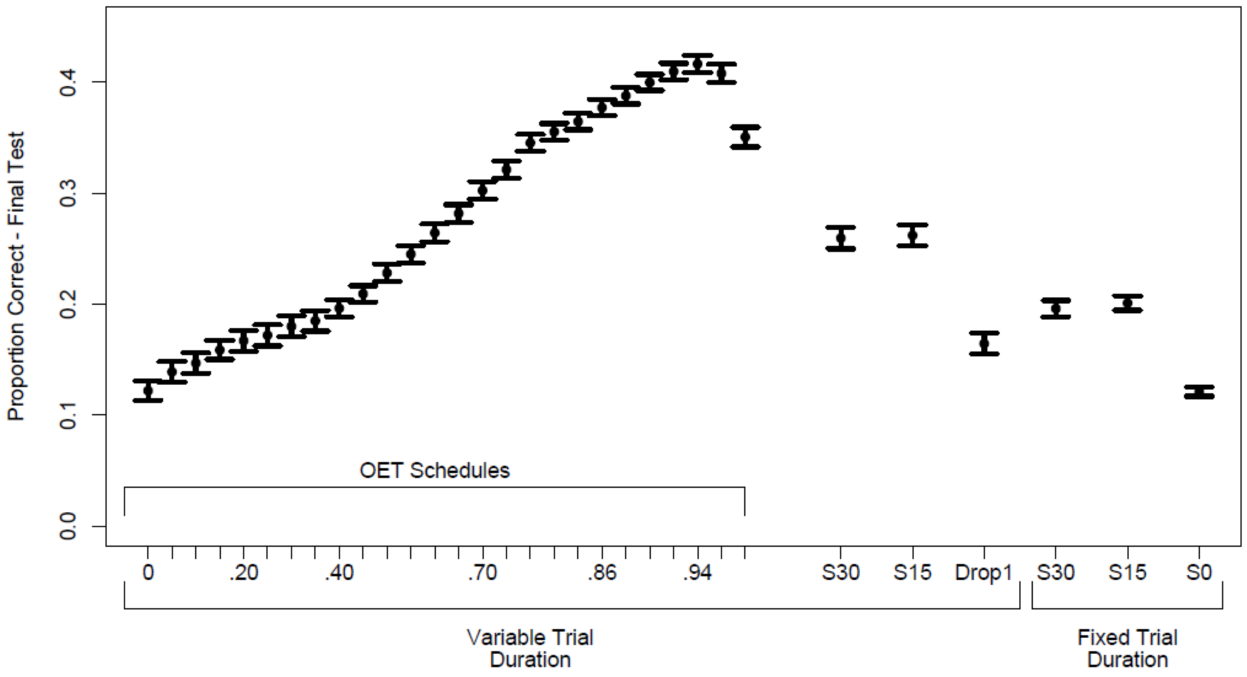

Supplementary Figure 2. Simulation results when correct response times are set to the median correct response time found in experimental data instead of using a latency model (as was described in the main text). i.e., one value of ~3 seconds was used for all correct trials in conditions with variable response times. See main text for description of how simulation was implemented. Errors bars represent +/- 1 SEM.
